# Assessing the relationship between epidemic growth scaling and epidemic size: The 2014–16 Ebola epidemic in West Africa

**DOI:** 10.1017/S0950268818002819

**Published:** 2018-10-15

**Authors:** Tapiwa Ganyani, Kimberlyn Roosa, Christel Faes, Niel Hens, Gerardo Chowell

**Affiliations:** 1 Interuniversity Institute for Biostatistics and statistical Bioinformatics, UHasselt (Hasselt University), Diepenbeek, Belgium; 2 Department of Population Health Sciences, School of Public Health, Georgia State University, Atlanta, GA, USA; 3 Centre for Health Economics Research and Modelling Infectious Diseases, Vaccine and Infectious Disease Institute, University of Antwerp, Antwerp, Belgium; 4 Division of International Epidemiology and Population Studies, Fogarty International Center, National Institute of Health, Bethesda, MD, USA

**Keywords:** Ebola epidemic, epidemic modeling, epidemic size, generalised growth model, sub-exponential growth

## Abstract

We assess the relationship between epidemic size and the scaling of epidemic growth of Ebola epidemics at the level of administrative areas during the 2014–16 Ebola epidemic in West Africa. For this purpose, we quantify growth scaling parameters from the ascending phase of Ebola outbreaks comprising at least 7 weeks of epidemic growth. We then study how these parameters are associated with observed epidemic sizes. For validation purposes, we also analyse two historic Ebola outbreaks. We find a high monotonic association between the scaling of epidemic growth parameter and the observed epidemic size. For example, scaling of growth parameters around 0.3–0.4, 0.4–0.6 and 0.6 are associated with epidemic sizes on the order of 350–460, 460–840 and 840–2500 cases, respectively. These results are not explained by differences in epidemic onset across affected areas. We also find the relationship between the scaling of epidemic growth parameter and the observed epidemic size to be consistent for two past Ebola outbreaks in Congo (1976) and Uganda (2000). Signature features of epidemic growth could become useful to assess the risk of observing a major epidemic outbreak, generate improved diseases forecasts and enhance the predictive power of epidemic models. Our results indicate that the epidemic growth scaling parameter is a useful indicator of epidemic size, which may have significant implications to guide control of Ebola outbreaks and possibly other infectious diseases.

## Introduction

When an infectious disease pathogen is spreading in a population, public health officials rely on indicators of epidemic growth to assess the risk of observing a major outbreak. Here, we argue that efforts to identify the signature features of epidemic growth could be utilised to assess the risk of observing a major outbreak, generate improved diseases forecasts and enhance the predictive power of epidemic models. These epidemic features are expected to vary across different infectious diseases in part influenced by the mode of transmission. For instance, Ebola is spread by direct contact via body fluids or indirect contact with contaminated surfaces. In contrast, influenza can be transmitted through the airborne route. For a given disease system, epidemic growth characteristics will vary across populations with different socio-demographic composition, resources for mitigating disease transmission, ethnicities and customs or traditions. While the growth profile of infectious disease outbreaks has been studied extensively assuming exponential growth, the growth dynamics have been shown to vary substantially across historic and contemporary epidemics of various diseases, including influenza, Ebola and HIV/AIDS [1–8]. For instance, the cumulative number of HIV/AIDS cases in the USA followed a cubic polynomial trajectory, likely as a result of population mixing mechanisms [4, 9]. Similarly, the Ebola epidemic in West Africa displayed varied polynomial (sub-exponential) growth patterns across affected administrative areas, whereas national aggregate incidence patterns appeared to follow exponential growth over short-time intervals [1, 10–12].

The above findings suggest that mathematical models of disease transmission need to incorporate flexible mechanisms that capture an appropriate range of the scaling of epidemic growth for the population and disease of interest [13–15]. The epidemic profile can be modulated by a combination of mechanisms and population characteristics including (1) heterogeneities in population structure stemming from social network patterns, (2) configurations of population susceptibility and infectiousness and (3) systematic changes in transmission rates over time as a result of changes in behaviour and/or the rapid implementation of public health interventions including cancellation of mass gatherings and school closings.

A number of factors influenced the dynamics of the 2014–16 Ebola outbreak including movement patterns often influenced by interventions (e.g. movement restrictions), use of quarantine for exposed individuals and unsafe funerals involving a large number of people [16]. The Ebola epidemics across administrative areas of Guinea, Liberia and Sierra Leone followed early sub-exponential growth rather than exponential growth over several generations of disease transmission [1]. The generalised growth model (GGM) [2] incorporates a scaling of growth parameter (*p*) in order to capture a range of epidemic growth patterns, ranging from constant growth, sub-exponential growth, to exponential growth. Essentially, a value of *p* closer to one indicates a higher threat level (more rapid growth) compared with smaller values. Thus, the GGM provides a useful tool to gain quantitative insights on the likely signature feature of the threat level. When applied to spatially disaggregated data, the model can, therefore, reveal variable transmission dynamics, which is useful for planning intervention measures (e.g. type and intensity of mitigation measures) as well as mobilising of resources. The 2-parameter GGM is given by the following differential equation [2]:
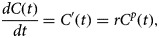
where *C*′(*t*) represents incidence at time *t*, *r* ϵ (0, ∞) is the growth rate per time unit and, *p* ϵ(0, 1) is the scaling of growth parameter that models constant incidence (*p* = 0), sub-exponential growth (0 *<* *p <* 1) and exponential growth dynamics (*p* = 1).

Empirical studies that link the scaling of growth of infectious disease outbreaks with epidemic size and then relate this relationship with underlying population characteristics, such as population density and urbanisation as well as response capacity, could shed light on key drivers of disease transmission and control across different pathogens and population settings. In this direction, we examine the variation in epidemic growth profiles of the devastating 2014–16 West African Ebola epidemic across administrative areas of the three most affected countries, namely Guinea, Liberia and Sierra Leone. We also quantify the relationship between the estimated scaling of growth parameter and the observed epidemic size across local outbreaks of Ebola.

## Materials and methods

### Data

A total of 28 610 cases and 11 308 deaths were reported during the 2014–16 Ebola epidemic in West Africa [17]. However, spatial heterogeneities in case burden and epidemic timing were observed across administrative areas [1]. For the three most affected countries, we retrieved the time-series of weekly cases of Ebola stratified at the level of administrative areas from the WHO Ebola patient database [17]. The data were collected and reported by national health authorities of Guinea, Liberia and Sierra Leone following national and/or WHO guidelines on case definitions for the period 5 January to 17 December 2014 [18]. In this study, we focus on characterising the early ascending phase of 24 sub-national Ebola outbreaks that comprise at least 7 weeks of epidemic growth. For these outbreaks, the number of weeks from onset to peak ranged from 7 to 21 weeks while the peak size of the weekly incidence curve ranged from 17 to 290 cases. Epidemic onset is defined as the week at which the period of sustained epidemic growth starts in each area. For validation purposes, we also analyse two sub-national Ebola outbreaks that occurred in Congo (1976), which affected the village of Yambuku [19] and in Uganda (2000), which mostly affected the district of Gulu [20].

### Parameter estimation

For each Ebola outbreak, we estimate parameters *r* and *p* by fitting the GGM to weekly early incidence growth data. Since the duration of onset-to-peak varied greatly among the study areas, we use onset-to-peak data so as to maximise usage of available data. It is worth noting that parameters can be estimated from the early phase (using data before peak) at the expense of quality of estimates. Indeed, other studies have shown that the uncertainty of parameter estimates improves when more data are used in fitting the model [2]. In sensitivity analyses, we also consider ascending phases from the onset until up to 1–3 weeks preceding the epidemic peak.

We estimate parameters *r* and *p* jointly through a maximum likelihood framework using an ordinary differential equation solver. Let *y*_*t*_ denote, in a given area, the case count at time *t*. Given the count nature of cases, we model *y*_*t*_ using a negative binomial (NB) distribution to incorporate the possibility of over-dispersion (variance of data greater than the mean),
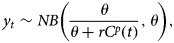
where θ ϵ (0, ∞) is the dispersion parameter, which we also estimate together with parameters *r* and *p*. Under this parameterisation we have that, *E*(*Y*_*t*_*|C*(*t*)) = *rC^p^*(*t*). The likelihood function is given by,
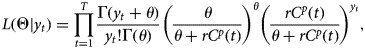
where Θ is the set of parameters: {*r*, *p*, *θ*} and, *T* is the length of the observed vector of case counts. The likelihood function can be maximised in a non-Bayesian or Bayesian framework. We perform Bayesian maximum likelihood estimation using the publicly available and free WinBUGS Differential Interface [21]. We assign minimally-informative priors to the parameters (supplement). The posterior distribution *P*(Θ|*y*_*t*_) is given by,
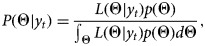
where *p*(Θ) represents the product of independent priors for {*r*, *p*, *θ*}. We obtain point estimates by calculating the mean of the converged posterior samples. The code is provided in the supplementary material.

### Characterising the association between observed epidemic size and *p*

The growth rate *r* in the GGM is an innocent parameter since it can be eliminated by rescaling time in the equation [22]. Hence, our focus is on the scaling of epidemic growth parameter *p*. To study the association between the observed epidemic size (denoted by *z*) and the estimated value of *p*, we conduct correlation and regression analyses. Note that the validation data are not used in studying the association between epidemic size and *p*. We use the index *i* to denote the *i^th^* area.

#### Spearman's rank correlation coefficient

The Spearman's rank correlation coefficient measures the strength and direction of the monotonic relationship between two variables, in this case between *z* and *p*. It is calculated as,
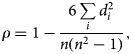
where *d*_*i*_ = rank(*z*_*i*_) – rank(*p*_*i*_). Its value lies between −1 and 1; the closer the magnitude of *ρ* to 1, the stronger the relationship indicating that the relationship between *z* and *p* can be described using a monotonic function [23]. We use the *spearman.ci*() function in *R* [24] to estimate *ρ* and its 95% confidence interval.

#### Negative binomial regression

Given the count nature of epidemic size, we also assume that *z*_*i*_ can be modelled as,
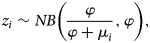
where, *φ* ϵ(0, ∞) is the dispersion parameter, *μ*_*i*_ = exp(*β*_*0*_ *+* *β*_1_*p_i_) and *p_**i**_* is the vector containing scaling of growth parameter values estimated across the administrative areas. Under this parameterisation we have that, *E*(*Z*_*i*_*|p_i_*) = *μ*_*i*_ and, *Var*(*Z*_*i*_*|p_i_*) = *μ*_*i*_ *+* *μ*^2^_*i*_/*φ*. Small values of *φ* indicate that the variance is greater than the mean (over-dispersion) and, *φ* = ∞ indicates that the mean and variance are equal (equi-dispersion). In this context, the parameter *φ* can be viewed as representing effects of unobserved variables or other sources of pure randomness that may determine the epidemic size (see e.g. [25]). Estimation proceeds in a Bayesian maximum likelihood framework as before, replacing *rC^p^*(*t*) by *μ*_*i*_ and taking Θ = {*β*_0_, *β*_1_, *φ*}. The posterior distribution is also evaluated in WinBUGS. We obtain point and interval estimates by summarizing converged posterior samples. The code is provided in the supplementary material.

## Results

For the 24 studied subnational Ebola outbreaks in West Africa and for the two validation outbreaks, our estimates of the scaling of growth parameter for four lengths of the ascending growth phase and the corresponding epidemic size are summarised in Table 1.

**Table 1.** Observed epidemic sizes for the 24 Ebola outbreaks; *p* estimates and 95% credible intervals obtained when the GGM is fitted, per area, for varying ascending phase lengths

Country	Area	Observed epidemic size	*p* estimates (95% credible interval) *at x weeks before peak*			
			0	1	2	3
Guinea	Conakry	548	0.873 (0.589–1.000)	0.638 (0.248–0.999)	0.595 (0.182–0.990)	0.389 (0.011–0.881)
Guinea	Forécariah	468	0.514 (0.333–0.682)	0.489 (0.284–0.670)	0.473 (0.263–0.664)	0.471 (0.248–0.694)
Guinea	Guéckédou	380	0.353 (0.184–0.511)	0.291 (0.150–0.432)	0.319 (0.159–0.467)	0.301 (0.155–0.460)
Guinea	Kérouané	140	0.421 (0.327–0.541)	0.404 (0.295–0.531)	0.531 (0.358–0.691)	0.622 (0.374–0.880)
Guinea	Kindia	110	0.485 (0.233–0.711)	0.344 (0.086–0.593)	0.467 (0.161–0.764)	0.445 (0.082–0.758)
Guinea	Macenta	702	0.772 (0.668–0.878)	0.798 (0.645–0.945)	0.933 (0.827–1.000)	0.803 (0.474–1.000)
Guinea	Nzérékoré	245	0.368 (0.287–0.454)	0.360 (0.279–0.449)	0.366 (0.275–0.462)	0.359 (0.267–0.472)
Sierra Leone	Bo	1498	0.773 (0.718–0.830)	0.794 (0.729–0.854)	0.797 (0.704–0.873)	0.771 (0.643–0.881)
Sierra Leone	Bombali	1070	0.886 (0.796–0.990)	0.930 (0.860–1.000)	0.972 (0.923–1.000)	0.949 (0.868–1.000)
Sierra Leone	Kailahun	741	0.521 (0.456–0.604)	0.528 (0.444–0.626)	0.548 (0.440–0.673)	0.571 (0.491–0.700)
Sierra Leone	Kambia	296	0.327 (0.179–0.479)	0.248 (0.111–0.379)	0.247 (0.106–0.390)	0.236 (0.105–0.391)
Sierra Leone	Kenema	537	0.457 (0.402–0.518)	0.459 (0.398–0.525)	0.469 (0.396–0.546)	0.484 (0.402–0.577)
Sierra Leone	Kono	564	0.527 (0.334–0.689)	0.450 (0.287–0.609)	0.461 (0.280–0.626)	0.418 (0.257–0.596)
Sierra Leone	Moyamba	297	0.545 (0.206–0.955)	0.160 (0.001–0.398)	0.230 (0.000–0.554)	0.121 (0.000–0.337)
Sierra Leone	Port Loko	2179	0.548 (0.500–0.598)	0.536 (0.480–0.588)	0.527 (0.466–0.581)	0.527 (0.465–0.592)
Sierra Leone	Tonkolili	617	0.920 (0.811–1.000)	0.739 (0.509–0.987)	0.791 (0.499–1.000)	0.367 (0.097–0.754)
Sierra Leone	Western rural	1711	0.561 (0.504–0.615)	0.565 (0.501–0.622)	0.581 (0.519–0.636)	0.601 (0.548–0.653)
Sierra Leone	Western urban	3152	0.595 (0.543–0.637)	0.574 (0.533–0.613)	0.582 (0.539–0.623)	0.577 (0.529–0.629)
Liberia	Bomi	195	0.315 (0.236–0.413)	0.297 (0.211–0.400)	0.317 (0.209–0.428)	0.376 (0.260–0.514)
Liberia	Bong	168	0.243 (0.140–0.372)	0.223 (0.103–0.350)	0.241 (0.120–0.378)	0.261 (0.128–0.409)
Liberia	Grand Cape Mount	139	0.406 (0.172–0.619)	0.205 (0.025–0.392)	0.227 (0.026–0.434)	0.241 (0.012–0.446)
Liberia	Lofa	465	0.451 (0.344–0.582)	0.408 (0.302–0.534)	0.378 (0.269–0.505)	0.361 (0.249–0.516)
Liberia	Margibi	830	0.805 (0.680–0.925)	0.836 (0.700–0.957)	0.868 (0.745–0.998)	0.893 (0.750–1.000)
Liberia	Montserrado	2683	0.821 (0.777–0.865)	0.835 (0.786–0.879)	0.844 (0.769–0.900)	0.831 (0.706–0.927)
Congo	Bumba	256	0.455 (0.313–0.597)	0.406 (0.252–0.564)	0.440 (0.261–0.588)	0.418 (0.253–0.600)
Uganda	Gulu	418	0.586 (0.500–0.670)	0.592 (0.482–0.693)	0.596 (0.458–0.722)	0.532 (0.390–0.695)

### Correlation analyses

Correlation analyses indicate a high monotonic association between the scaling of growth parameter and the observed epidemic size (Table 2) – specifically, the greater the estimated value of *p*, the greater the observed epidemic size.

**Table 2.** Spearman correlation coefficient (*ρ*) between the scaling of epidemic growth parameter *p* and the observed epidemic size (*z*) calculated using *p* estimates from varying ascending phase lengths

Weeks before peak	*ρ*	95% confidence interval
0	0.756	(0.548–0.854)
1	0.820	(0.633–0.903)
2	0.746	(0.507–0.875)
3	0.673	(0.332–0.867)

The strength of the association appears to decline as the number of data points comprising the ascending phase decreases below the epidemic peak. This is to be expected as the quality of *p* estimates can be affected by the amount of available data as well as the quality of information contained in the data. Hence, this association tends to be weaker as the number of data points of the ascending phase is reduced due to biased and/or high-uncertainty estimates of the scaling of the growth parameter.

### Regression analyses

**Fig. 1.**
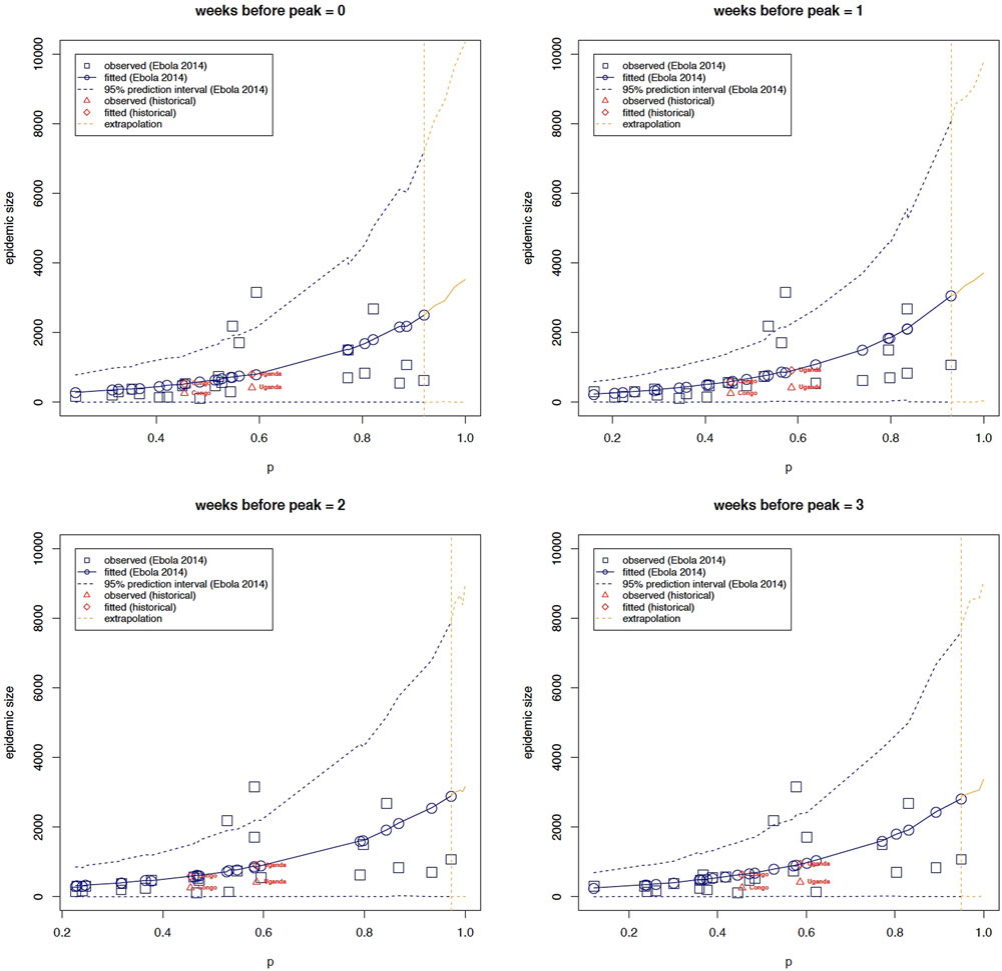
The relationship between epidemic size and the scaling of epidemic growth parameter *p* across 24 administrative-level Ebola outbreaks comprising at least 7 weeks of epidemic growth, for varying ascending phase lengths. The relationship between epidemic size and scaling of epidemic growth parameter is consistent for two past Ebola outbreaks that occurred in Congo in 1976, which affected the village of Yambuku [18] and in Uganda (2000), which mostly affected the district of Gulu [19]. The relationship was extrapolated from our highest estimate of *p* (around 0.93; vertical dashed line) to *p* = 1.

Table 3 shows parameter estimates obtained when observed epidemic size is regressed on *p* using estimates derived for different lengths of the ascending outbreak phase as explained above. These results are in-line with correlation analyses – areas with greater deceleration parameters have greater epidemic sizes and vice-versa. More specifically, e.g. using onset to peak data, for a *δ* unit increase in *p*, the expected epidemic size increases by a multiplicative factor of exp(δ*β_*1*_) = exp(δ*3.201) with a corresponding confidence given by (exp(δ*1.119), exp(δ*5.249)). In other words, denoting the expected epidemic size of a deceleration parameter equal to *p* by *z*_*p*_, the expected epidemic size of a deceleration parameter equal to *p* + *δ* (*z*_*p+δ*_) equals the product of exp(δ*3.201) and z_*p*_ (Fig. 1). As already observed in the correlation analyses, this positive effect appears to decline as the number of data points are reduced.

**Table 3.** Parameter estimates of the NB regression model: observed epidemic size is regressed on the scaling of epidemic growth parameter *p* using data from varying ascending phase lengths

Weeks before peak	Effect	Estimate (95% credible interval)
0	*β*_*1*_	3.201 (1.119–5.249)
	*φ*	1.670 (0.820–2.600)
1	*β*_*1*_	3.267 (1.797–4.862)
	*φ*	2.097 (1.028–3.197)
2	*β*_*1*_	2.925 (1.378–4.746)
	*φ*	1.780 (0.968–2.841)
3	*β*_*1*_	2.941 (1.322–4.583)
	*φ*	1.874 (0.973–2.927)

Our negative binomial regression analyses indicate significant over-dispersion (*φ*) within the data. Indeed, since *p* is estimated it is prone to pure randomness especially during the early transmission stages. Moreover, it is prone to possibly non-random variability that is unobservable, observable and quantifiable, or, observable but difficult to quantify (*φ* represents random and/or non-random heterogeneity). Indeed, several factors are understood to have shaped the dynamics of the 2014 West Africa Ebola outbreak, e.g. intervention measures, under-reporting, delayed reporting, environmental factors (see, e.g. [26]).

Overall, there is a positive monotonic relationship between observed epidemic size and the estimated growth scaling parameter, indicating the parameter *p* is predictive of epidemic size. Moreover, the regression model predicts epidemic size for the historical outbreaks in Uganda (2000) and Congo (1975) well within the 95% prediction interval (Fig. 1).

## Discussion

In this paper, we analyse the relationship between observed epidemic size and epidemic growth scaling of major Ebola outbreaks at the subnational level of the three most affected countries during the devastating 2014–16 Ebola epidemic in West Africa. We find a statistically significant relationship between epidemic size and scaling of epidemic growth parameter. Specifically, when an epidemic shows sustained growth, the expected epidemic size is exponentially related to scaling of growth – epidemics with lower scaling of growth have smaller epidemic sizes and vice-versa. For example, scaling of growth parameters around *p* = 0.3–0.4, *p* = 0.4–0.6 and *p* > 0.6 were associated with epidemic sizes on the order of 350–460, 460–840 and 840–2500 cases, respectively. Moreover, the timing of epidemic onset (calendar week at which epidemic starts to show sustained growth) did not explain differences in epidemic growth scaling across affected areas.

We find that this relationship also holds for two past Ebola outbreaks in Congo (1976) and Uganda (2000). Specifically, our model calibrated using data from the 2014–16 Ebola epidemic in West Africa predicts the expected epidemic sizes to be approximately equal to 541 and 824, against observed values equal to 256 and 418, respectively. Note that our model predictions are affected by some outlying data points, hence the overestimation. Predictions closer to observed values are obtained when some outlying data points are excluded from the model calibration stage (see supplement). It is also worth noting that an Ebola outbreak recently occurred in the Democratic Republic of Congo, however, the number of cases is rather limited or the epidemic growth phase is very short [27].

Empirical studies characterising the relationship between the scaling of growth parameter and final epidemic size are needed for diseases beyond Ebola and should connect this relationship to underlying population characteristics such as population density, urbanisation as well as local capacity to prevent or mitigate the spread of diseases. Indeed, in the context of the 2014–16 Ebola epidemic, prior studies have suggested the role of population density and its impact on transmission potential [28] and outbreak size [5, 29]. More broadly, our results suggest that epidemic growth indicators could contain useful information to characterise epidemic growth dynamics and their likely epidemic size. These results also point to the need to design mathematical models that incorporate characteristics of the epidemic growth dynamics, which is likely to enhance forecasting and predictive power of mathematical models. In fact, models that incorporate the generalised-growth dynamics have shown promising forecasting performance when confronted against real and synthetic epidemics [3, 7, 8].

Our results also confirm the spatial heterogeneity of growth patterns of the 2014–16 Ebola epidemic in West Africa ranging from very slow to nearly-exponential growth [1]. We have previously noted that epidemic incidence patterns encompassing large spatial scales (e.g. national) can mask substantial heterogeneities that are only evident at smaller spatial scales (e.g. county or administrative area) [1]. During the 2014–16 Ebola epidemic, national incidence curves for Guinea, Sierra Leone and Liberia displayed short-lived exponential growth periods. Yet, local epidemics at the level of administrative areas were asynchronous and early incidence growth largely followed polynomial dynamics during 3–4 generations of disease transmission [1]. It is also worth pointing out that epidemic growth patterns are partially influenced by intrinsic factors relating to the natural history of the disease (e.g. transmission mode, the variability of the incubation period) [30] and the particular characteristics of the geographic setting where the epidemic takes place [6].

In summary, our findings indicate that the generalised growth model is convenient and advantageous in characterising differences in patterns of epidemic growth scaling, which we found to be statistically related to epidemic size. Our results suggest that the epidemic growth scaling parameter is a useful indicator of epidemic impact, which may have significant implications to guide outbreak control for Ebola and possibly for other infectious diseases. Our results should motivate further studies to characterise differences in growth scaling across different disease systems and how these and other local factors drive epidemic size. From a public health perspective, our study could have significant implications for informing control interventions and public health resources allocation, e.g. prioritise control in areas that exhibit exponential or near-exponential growth dynamics.

## Supplementary material

For supplementary material accompanying this paper visit https://doi.org/10.1017/S0950268818002819. click here to view supplementary material
