# Supplementary material for: Assessing the relationship between epidemic growth scaling and epidemic size: The 2014–16 Ebola epidemic in West Africa
Source: Epidemiol Infect. 2018 Oct 15;147:e27. doi: 10.1017/S0950268818002819 (PMC6518536; doi:10.1017/S0950268818002819)
Supplement: Supplementary file 1 [file S0950268818002819sup001.docx]

**Supplementary material**

**WinBUGS code for fitting the GGM to a time series**

model{

solution[1:ngrid, 1:ndim] <- ode(init[1:ndim], tgrid[1:ngrid], D(C[1:ndim], t), origin, tol)

D(C[I], t) <- rweek*pow(C[I], p)

init[I] <- initcase

sol[1] <- solution[1, 1]

for(k in 2:ngrid){

sol[k] <- solution[k, 1] - solution[(k-1), 1]

}

for (i in 2:ngrid){

cases[i] ~ dnegbin(mu[i], theta)

mu[i] <- theta/(theta+sol[i])

}

p ~ dunif(0,1)

rweek ~ dunif(0,5)

theta ~ dgamma(0.001, 0.001)

r <- rweek*(1/7)

}

**WinBUGS code for fitting negative binomial regression**

model{

for (i in 1:N){

sol[i] <- exp(beta[1] + beta[2]*p[i])

mu[i] <- phi /(phi+sol[i])

y[i] ~ dnegbin(mu[i], phi)

}

phi ~ dgamma(0.001, 0.001)

for(k in 1:2){

beta[k] ~ dnorm(0, 0.0001)

}

}

**Exploring the sensitivity of results to the removal of outlying cases**

Here, we present similar analyses as presented in the main document after removing four observations which are outlying in the observed epidemic sizes (the highest 4). Tables 2a and 3a correspond to Tables 2 and 3 in the main document, respectively.

Table 2a Spearman correlation coefficient (ρ) between the deceleration parameter p and the observed epidemic size (z) calculated using p estimates from varying ascending phase lengths excluding outlying cases.

| **weeks before peak** | **𝛒** | **95% confidence interval** |
| --- | --- | --- |
| 0 | 0.762 | (0.526, 0.886) |
| 1 | 0.854 | (0.588, 0.941) |
| 2 | 0.773 | (0.421, 0.929) |
| 3 | 0.624 | (0.179, 0.876) |

Table 3a Parameter estimates of the NB regression model: observed epidemic size is regressed on the deceleration parameter p using data from varying ascending phase lengths excluding outlying cases.

| **weeks before peak** | **Effect** | **estimate (95% credible interval)** |
| --- | --- | --- |
| 0 | β*_1_* | 2.476 (1.284, 3.611) |
|  | *φ* | 2.108 (1.872, 7.065) |
| 1 | β*_1_* | 1.578 (1.552, 3.297) |
|  | *φ* | 2.763 (2.418, 9.654) |
| 2 | β*_1_* | 2.925 (1.227, 3.122) |
|  | *φ* | 2.144 (1.913, 7.359) |
| 3 | β*_1_* | 1.990 (0.848, 3.030) |
|  | *φ* | 3.681 (1.637, 6.211) |


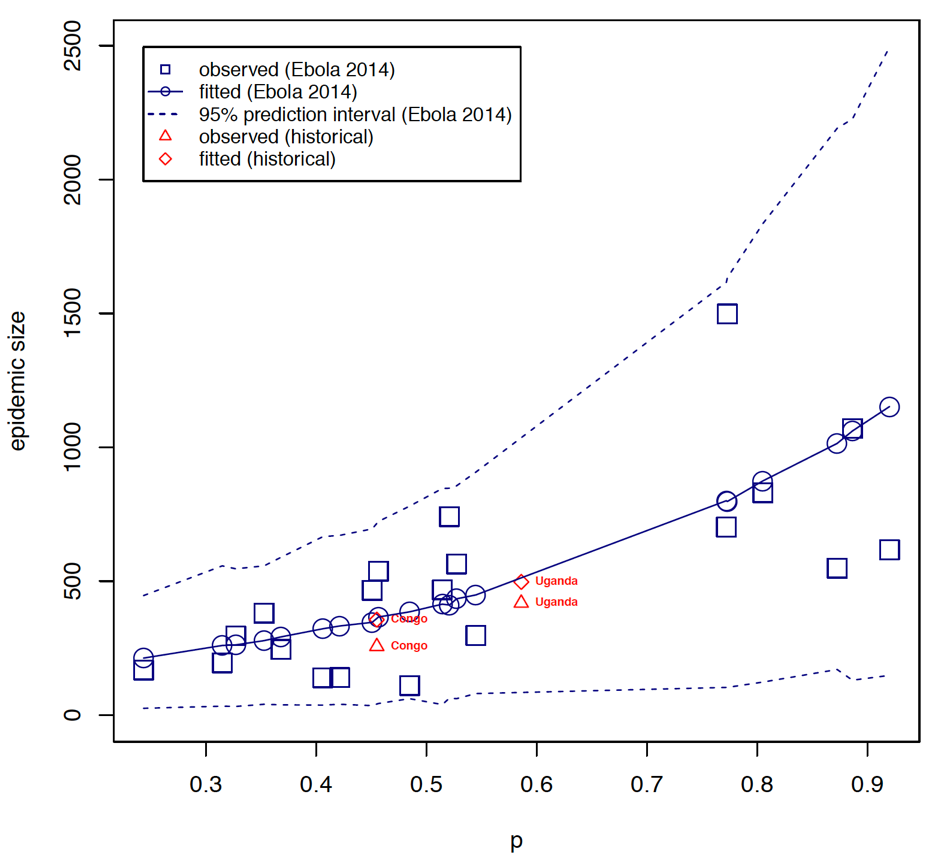


Figure 1a The relationship between epidemic size and the scaling of epidemic growth parameter p across 24 administrative-level Ebola outbreaks comprising at least 7 weeks of epidemic growth (excluding outliers). Here we display estimates of p derived using data of the ascending growth phase (from epidemic onset to peak). The relationship between epidemic size and scaling of epidemic growth parameter is consistent for two past Ebola outbreaks that occurred in Congo in 1976), which affected the village of Yambuku and in Uganda (2000), which mostly affected the district of Gulu.

For the two past Ebola outbreaks in Congo (1976) and Uganda (2000), our model predicts the expected epidemic sizes to be approximately equal to 356 and 497, against observed values equal to 256 and 418, respectively.
